# Sterile Neuroinflammation and Strategies for Therapeutic Intervention

**DOI:** 10.1155/2017/8385961

**Published:** 2017-01-03

**Authors:** Manoj Banjara, Chaitali Ghosh

**Affiliations:** ^1^Cerebrovascular Research, Cleveland Clinic Lerner Research Institute, Cleveland Clinic, Cleveland, OH, USA; ^2^Department of Biomedical Engineering, Cleveland Clinic Lerner Research Institute, Cleveland Clinic, Cleveland, OH, USA; ^3^Department of Molecular Medicine, Cleveland Clinic Lerner Research Institute, Cleveland Clinic, Cleveland, OH, USA

## Abstract

Sterile neuroinflammation is essential for the proper brain development and tissue repair. However, uncontrolled neuroinflammation plays a major role in the pathogenesis of various disease processes. The endogenous intracellular molecules so called damage-associated molecular patterns or alarmins or damage signals that are released by activated or necrotic cells are thought to play a crucial role in initiating an immune response. Sterile inflammatory response that occurs in Alzheimer's disease (AD), Parkinson's disease (PD), stroke, hemorrhage, epilepsy, or traumatic brain injury (TBI) creates a vicious cycle of unrestrained inflammation, driving progressive neurodegeneration. Neuroinflammation is a key mechanism in the progression (e.g., AD and PD) or secondary injury development (e.g., stroke, hemorrhage, stress, and TBI) of multiple brain conditions. Hence, it provides an opportunity for the therapeutic intervention to prevent progressive tissue damage and loss of function. The key for developing anti-neuroinflammatory treatment is to minimize the detrimental and neurotoxic effects of inflammation while promoting the beneficial and neurotropic effects, thereby creating ideal conditions for regeneration and repair. This review outlines how inflammation is involved in the pathogenesis of major nonpathogenic neuroinflammatory conditions and discusses the complex response of glial cells to damage signals. In addition, emerging experimental anti-neuroinflammatory drug treatment strategies are discussed.

## 1. Background

Inflammation is a response of the innate immune system that is triggered by infection or injury. It aims to protect and defend the body by clearing and controlling the initial stimulus, through the release of cells and mediators that combat foreign substances and thereby help to prevent infection [1]. Even though inflammation is intended to be protective and beneficial, an excessive inflammatory response can cause further tissue damage. Once activated, primed inflammatory cells may target remote sites, indicating detrimental effects of long-term inflammation [2].

For decades, brain has been considered as an immune privileged site due to the presence of highly restrictive blood-brain barrier (BBB). However, “neuroinflammation,” inflammation of the central nervous system (CNS), does occur. Neuroinflammation is evident in various CNS disorders including Alzheimer's disease (AD), Parkinson's disease (PD), Huntington's disease (HD), amyotrophic lateral sclerosis (ALS), stroke, epilepsy, and traumatic brain injury (TBI). The inflammatory triggers in these conditions are the endogenous damage-associated molecular patterns (DAMPs) in the absence of infection.

The focus of this review is to understand sterile or non-pathogen-associated neuroinflammation and its players in multiple CNS disorders. The evidences gathered here suggest that neuroinflammation causes and accelerates neurodegeneration and vice versa. Further, there are common players and pathways of neuroinflammation in these inflammatory brain diseases. Here we aim to identify and discuss on the anti-neuroinflammatory drug target strategies that may specifically target DAMPs-induced inflammation in brain but not globally suppress the immune system.

## 2. Neuroinflammation

**Figure 1:**
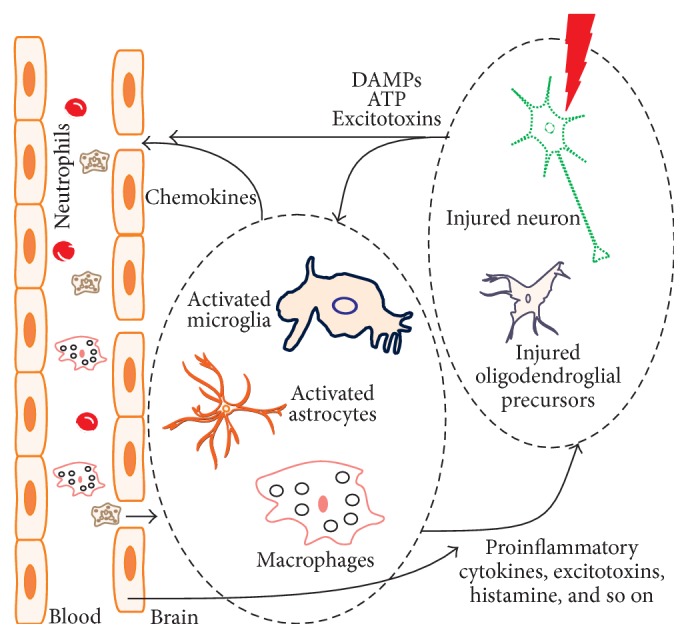
Scheme of early innate response to brain injury. Damage signals or DAMPs primarily released from the injured parenchymal cells are sensed by immune effector cells such as microglia, astrocytes, and macrophages. The triggered innate immune response (e.g., proinflammatory cytokines, chemokines, reactive oxygen species, excitotoxins, histamine, and prostaglandins) has detrimental influences on the neurons, oligodendroglial precursors, and vascular endothelial cells. The increased BBB permeability contributes the migration of peripheral immune cells (e.g., neutrophils, mast cells, and macrophages) to the sites of tissue damage.

Neuroinflammation describes the broad range of immune responses of the CNS, which could be initiated in the periphery or within the brain. The proinflammatory mediators derived from the peripheral inflammation can release and transmit these mediators and permit migration of leucocytes into the brain (Figure 1) [2, 3]. The entry of peripheral leukocytes by BBB damage (BBBD) creates a scenario similar to that seen in peripheral inflammatory response. In addition, exacerbation of brain damage causes neuronal injury, triggering neuroinflammatory responses [2, 4]. Thus, even in the absence of peripheral infiltration of immune cells, nervous system undergoes constant immune surveillance by the resident brain cells, primarily microglia and astrocytes [2].

**Figure 2:**
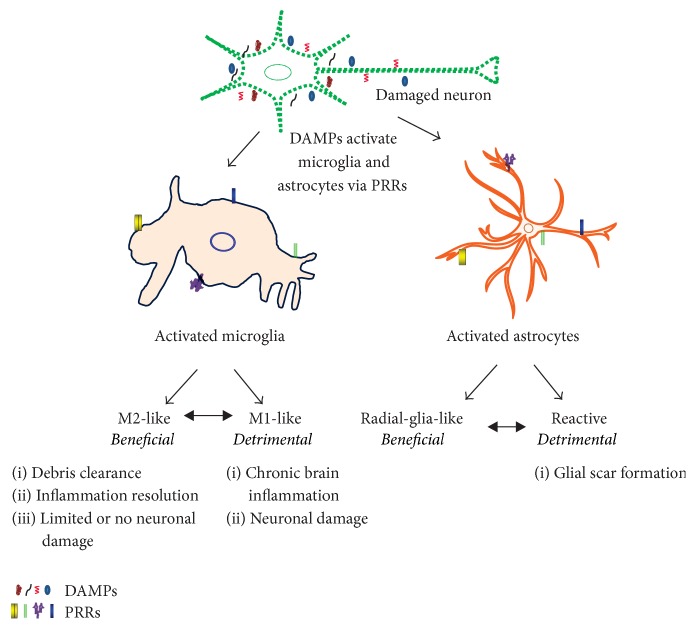
Response of microglia and astrocytes to the brain injury. DAMPs can signal PRRs expressed in astrocytes and microglia, promoting their activation. Depending on the injury site, severity of brain injury, surrounding environment, and signaling strength, astrocytes and microglia may respond to remove stimulants or to secrete inflammatory mediators. Typically, beneficial activation (M2-like microglia and radial-glia-like astrocytes) is associated with the elevated release of neurotrophic factors, anti-inflammatory cytokines (e.g., IL-4 and IL-10), and enzymes (e.g., arginase 1 and insulin-degrading enzymes) that enhance phagocytic activity. Conversely, detrimental activation of astrocytes and microglia is associated with the elevated and sustained expression of inducible nitric oxide synthase, reactive oxygen species, proinflammatory mediators (e.g., IL-1*α*/*β*, IL-6, and TNF), and decreased secretion of neurotrophic factors. These divergent responses may determine whether microglia and astrocytes lead to clear tissue debris or promote chronic neuroinflammation.

Brain cells express specialized pattern-recognition receptors (PRRs) that garnered increasing attention, as they are capable of triggering inflammatory pathways. These PRRs can sense microbial molecules, termed pathogen-associated molecular patters (PAMPs), or host-derived endogenous molecules, so-called danger/damage-associated molecular patterns (DAMPs). PAMPs are foreign molecules typically accumulated in the infected tissues, whereas DAMPs are misfolded proteins, aggregated peptides, or mislocalized nucleic acids found in diseased brain (Figure 2).

In the normal situation, neuroinflammation is a cellular and molecular response that aims to clear pathogens and dead/damaged cells generated by infection or injury and assist in returning the compromised area back to normal state. Hence, neuroinflammation is beneficial as it may sounds but has also been implicated in many CNS diseases. The persistent release of proinflammatory mediators diverts immune competent cells from the beneficial “housekeeping” functions to the detrimental “neurodegenerative” conditions [5, 6].

**Figure 3:**
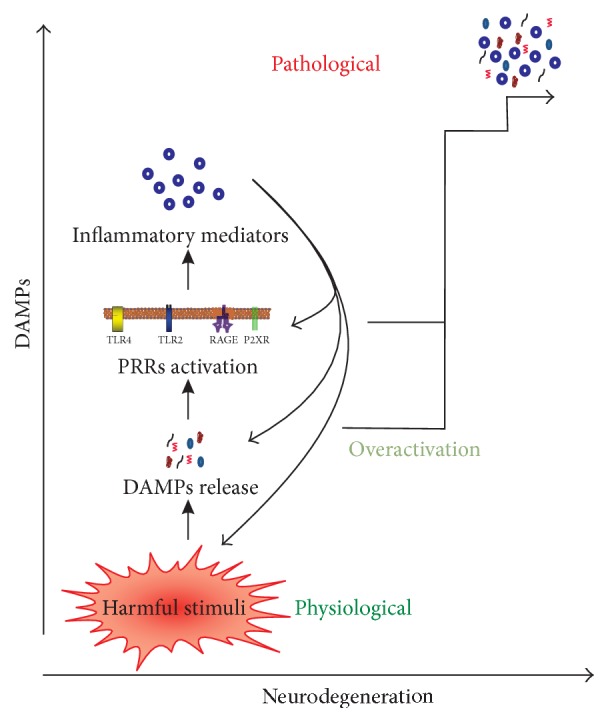
Propagation of “damage signals.” Harmful stimuli in the brain (e.g., brain injury and excessive neurodegeneration) generate endogenous DAMPs that induce the release of inflammatory mediators by activating PRRs. In turn, these molecules upregulate their own expression, directly activate the release of DAMPs, and trigger further tissue damage leading to increasing DAMPs level. Hence, a sustained aggressive cycle may result in chronic neuroinflammation. However, a controlled release of DAMPs has beneficial roles in immunity and tissue repair process.

**Figure 4:**
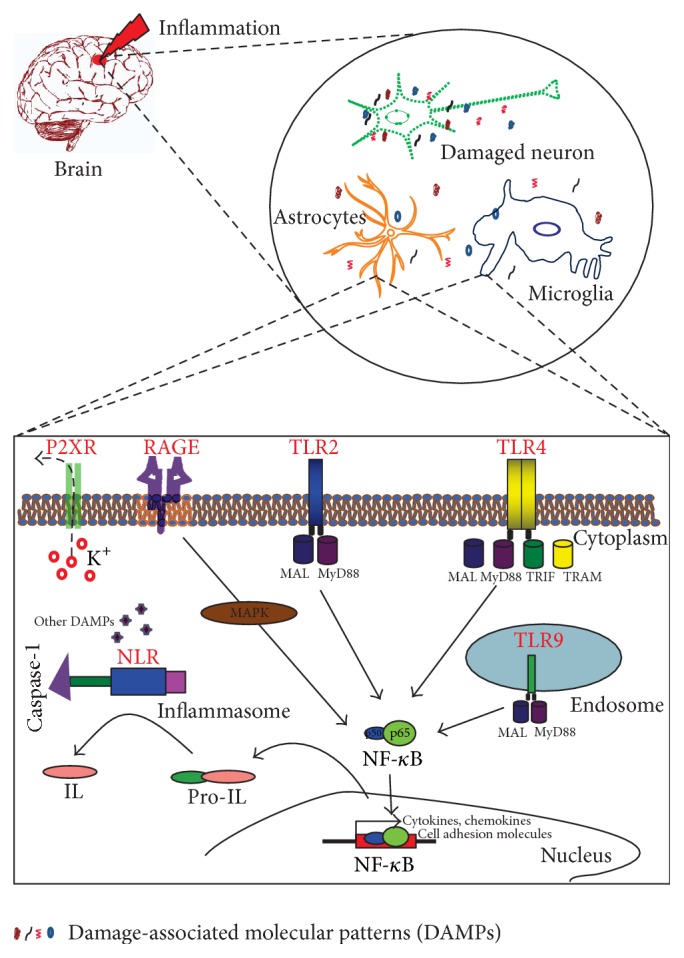
Mechanisms of glial cell activation in response to damage signals. Sterile neuroinflammatory conditions are characterized by the accumulation of misfolded and aggregated proteins in the brain. These DAMPs are released from different subcellular components of the damaged neurons, which trigger respective PRRs leading to downstream activation of proinflammatory cascades and enhancing effects of initial inflammatory insult. Activation of PRRs, primarily TLR2, TLR4, TLR9, and RAGE, converge largely into NF-*κ*B activation, promoting cell death and/or contributing to neuroinflammatory/neurodegenerative mechanisms. These pathways, including P2XR, jointly work with multiprotein inflammasome complex (NLRs) that assists the generation of mature cytokines from proforms via the activation of caspase-1. TLR, toll-like receptor; RAGE, receptor for advanced glycation end products; NLR, nod-like receptor; P2XR, ATP-gated purinergic P2 receptors; MyD88, myeloid differentiation primary response gene (88); MAL, MyD88-adapter-like; TRIF, TIR-domain-containing adapter-inducing interferon-*β*; TRAM, TRIF-related adaptor molecule; MAPK, mitogen-activated protein kinase; NF-*κ*B, nuclear factor-kappa B; IL, interleukin.

Acute neuroinflammation refers to the inflammatory reactions occur immediately after CNS injury, in which BBB is generally intact. In the absence of BBB breakdown after brain injury, its own immune system, largely composed of glial cells, are the subtle responders. The neuronal insults trigger glial activation without breaking BBB and concomitant infiltration of leukocytes. In the context of understanding CNS diseases, the concept of chronic inflammation is very relevant, as the term “disease” implies “chronicity.” The persistent neuroinflammation can be triggered by the infection of non-self-substances (e.g., pathogens and toxins) or endogenous molecules. Infections are classically accepted as inflammatory in nature, with meningeal, perivascular, or parenchymal infiltration of peripheral leukocytes [7]. However, there are some conditions that develop extreme neuroinflammation in the absence of notable peripheral infiltration including rabies, human immunodeficiency virus (HIV) infection, and prions disease. Studies suggest that HIV and prion infections alter microglial physiology, which is likely to initiate neurodegeneration that could contribute to the development of dementia occurring in these conditions [7]. Inflammation of CNS in the absence of pathogens and toxins has been termed sterile neuroinflammation (Figures 1–4). Some of the principal neuroinflammatory disorders are discussed below.

### 2.1. Traumatic Brain Injury

Within minutes of a traumatic effect, a robust inflammatory response is initiated in the injured brain. This posttraumatic squeal involves the activation of resident glial cells (microglia and astrocytes) and the infiltration of blood leukocytes. In addition, cytokines (e.g., IL-1, TNF, and IL-6) and chemokines (MCP-1, MIP-1, and RANTES) drive the accumulation of parenchymal and peripheral immune cells in the injured brain regions [8]. The humoral immune response is particularly seen in the acute phase following TBI, whereas the activation of glial cells appears to be sustained for several months [8]. In animal models of focal and diffuse TBI, IL-1*β* increased from the very low basal levels to detectable levels as early as 1 hr after trauma [9, 10]. IL-1*β* is hardly detectable in the cerebrospinal fluid (CSF) or serum of healthy individuals; thus, it is hard to measure following human TBI [11]. However, a study of postmortem tissue from TBI patients has confirmed the global elevation of IL-1*β* within a few minutes to hours after injury [12].

### 2.2. Epilepsy

Experimental and clinical findings support an important role of inflammation in the mechanisms underlying the generation of seizures [13]. Rodent studies demonstrate that seizures induce high levels of inflammatory mediators in brain regions that are involved in the generation and propagation of epileptic activities [14–17]. Proinflammatory cytokines (e.g., IL-6, IL-1*β*, and TNF-*α*) are upregulated in activated astrocytes and microglia that trigger a cascade of inflammatory events, involving neurons and vascular endothelial cells. More specifically, inflammatory cytokines activate multiple pathways such as NF-*κ*B, cyclooxygenase-2 (COX-2), complement system, chemokines, and acute phase proteins [17–19]. The rapid release of DAMPs from neurons, astrocytes, and microglia following proconvulsant injuries and activation of toll-like receptors (TLRs) in astrocytes and neurons is considered as a crucial event for initiating brain inflammation [20, 21]. In seizure models, brain inflammation is thought to be elevated by BBB breakdown via the disruption of tight-junction organization [22–24]. In human epilepsy, activation of both innate and adaptive immune systems has been described clearly. The analysis of epileptogenic tissue showed upregulation of high-mobility group box  1 protein (HMGB1) and IL-1*β* and their receptors, TLR4, receptor for advanced glycation end products (RAGE), and IL-1R, in glial cells and neurons [20, 25–29].

### 2.3. Stroke

Hypoxia and energy deficiency cause instantaneous cellular injury or death. The activation of microglia was seen in the penumbra after the first hour to days of ischemic event [30, 31]. Large number of reports and evidences directly link inflammatory reactions with the degree of stroke-associated brain damage and infarct growth. In addition, inflammation mediators, infarct size, and brain edema were markedly reduced by anti-inflammatory treatments [30, 32]. The activation of innate immune responses has key role in the generation of proinflammatory molecules. The release of DAMPs such as HMGB1 by neurons (passive) and astrocytes (active transport) was detected as early as 6 hr after onset until day 21 after stroke [33]. Further, other DAMPs such as heat shock proteins (HSPs) and adenosine triphosphate (ATP) are thought to be released from dying cerebral tissue after stroke that are sensed by putative receptors (e.g., TLR2, TLR4, and RAGE) to signal mitogen-activated protein kinases (MAPKs) and nuclear factor-kappa B (NF-*κ*B) resulting the stimulation of inflammatory cascades, leading to the expression of TNF-*α*, IL-1*β*, ICAM-1, VCAM-1, E-selection, and iNOS [30].

### 2.4. Psychological Stress

Innate immune responses are now thought to be a major etiology of numerous psychiatric disorders including posttraumatic stress disorder (PTSD), depression, and bipolar disorder [34, 35]. The experience of life stressors is a predisposing factor in the development of psychological disorders, which seems entirely unrelated to neuroinflammation. However, clinical reports indicate that stress predisposes individuals to inflammatory disorders (e.g., cardiovascular disease [36]), having high comorbidity with psychiatric conditions (e.g., depression) [37]. Acute exposure to stressor induces a rapid increase of proinflammatory cytokines in stress-reactive areas of the brain such as hypothalamus and hippocampus [38]. At least in part, the rapid increase of IL-1*β* expression in glial cells is due to the release of norepinephrine in response to stressful events [39]. More recent evidence implicated that HMGB1 is a stress signal to prime microglia for the expression of proinflammatory mediators in the brain. Blocking of TLR2 and TLR4 prevented neuroinflammatory responses during stress exposure which further supported the notion of neuroinflammation during psychological stress [40].

### 2.5. Alzheimer's Disease

In AD, microglia and astrocytes were reported to localize to amyloid plaques. Hence, neuroinflammation has been implicated in the pathology of AD [41–44]. Even though it is clear that not all microglial activation is deleterious to neurons, it is widely accepted that chronic activation of a microglial phenotype plays major role in the pathophysiology of AD [43]. Microglia and astrocytes in and around A*β* plaques release proinflammatory factors and proteases, suggesting innate immune response is a major contributor to plaque-induced toxicity [45]. Per se, TLR4 and RAGE have been suggested as a major mediator of AD [46].

### 2.6. Parkinson's Disease

The pathological hallmark of PD is the presence of *α*-synuclein-positive inclusions in the cell body (Lewy bodies) and processes (Lewy neurites) of specific neurons of the brain stem. In addition, a classic motor phenotype resulting from substantial loss of dopaminergic neurons from the substantia nigra pars compacta (SNPC) is evident in PD (review [47]). The presence of inflammatory mediators such as TNF, IL-1*β*, IL-6, and IFN*γ* in the cerebrospinal fluid and postmortem SNPC of PD patient confirmed the association between neuroinflammation and PD [43, 48, 49].

### 2.7. Huntington's Disease

Huntington's disease (HD) is an autosomal dominant neurodegenerative disorder that is associated with mutations in the huntingtin gene (htt) [50]. During HD and HD-like pathology, inflammation occurs in the CNS, increasing gliosis and expression of inflammation-related genes, including GFAP and complement proteins [51]. Expression of mutant “htt” in microglia itself is enough to increase the expression of proinflammatory genes such as TNF-*α* and IL-6 [52]. The proinflammatory signals are thought to stimulate microglia further in inducing neuronal death, and this, in turn, could lead to the activation of chronic “feed-forward loop” as shown in Figure 3 [53].

### 2.8. Amyotrophic Lateral Sclerosis

ALS is associated with a progressive degeneration of motor neurons in the CNS. Most ALS cases are sporadic in origin; however, 5–10% cases are caused by an autosomal dominant mutation. It is generally fatal within 5 yr of diagnosis due to a progressive generalized paralysis, weakening respiratory muscles, and causing respiratory failure. In ALS patients and mouse models of ALS, areas with degenerating motor neurons are marked by the presence of abundant cytokines (e.g., TNF, MCP-1, TGF-*β*, and IFN-*γ*) and inflammatory cells (e.g., T cells, activated microglia, and astrocytes) [54–56].

## 3. Components of Neuroinflammation

Brain injury and neurodegeneration are characterized by the increase in amount of proinflammatory cytokines and numbers of activated microglia [57]. Since inflammation is a key pathological change observed in these conditions [58], it is a valuable therapeutic target in the treatment of brain injuries and neurodegeneration. Microglia and astrocytes are highly specialized to detect and respond neuronal health and activity. However, endothelial cells also respond and release a large number of inflammatory mediators (Figures 1 and 2).

The innate immune system triggers inflammatory and regulatory responses via PRRs, complement system, cytokines, and chemokines in order to counteract brain injury and maintenance of tissue homeostasis. A large number of stimulators, their receptors, transcription factors, and intermediate molecules are involved for the expression of cytokines and chemokines that has key role in the regulation of neuroinflammation. A recent review article by Ransohoff in science suggested the higher levels of inflammatory mediators in parenchyma of stroke and TBI compared to AD, PD, and ALS, indicating potential roles of environmental triggers in the latter group [6]. Here, we explicitly discuss major players involved in neuroinflammation.

### 3.1. Microglia

Microglia is the resident macrophage of the CNS that is ubiquitously distributed in brain. Microglia constantly survey assigned regions in the brain using their highly motile processes, for the presence of pathogenic molecules and endogenous debris. Simultaneously, microglia provide factors to support tissue maintenance [59], protection and remodeling of synapses, and maintenance and plasticity of neuronal circuits [60]. Microglia activated by the pathological triggers (e.g., neuronal debris and protein aggregates) extend its processes to the injury site or migrate to the lesion, where they initiate an innate immune response (Figures 1 and 2) [2]. Such pathological triggers are recognized by receptors that are discussed in later sections.

In response to signaling molecules like DAMPs and cytokines, microglia transform from inactive (ramified) to active (phagocytic) state, releasing more proinflammatory molecules (Figures 1 and 2). In chronic neuroinflammation, these cells are activated for extended periods, releasing large amount of cytokines and neurotoxic molecules that contribute to neurodegeneration [61]. Macrophage activation can be categorized as M1 (classically activated) or M2 (alternatively activated). M1 macrophages are effector macrophages that are stimulated by IFN*γ* and TNF to produce aggressive first-line immune response. M2 represents other types of macrophages, usually stimulated by IL-4, having anti-inflammatory roles in wound healing and macrophage response regulation [2]. The switching of M2 to M1 state is thought to have significant effect on the intensity and development of peripheral inflammation (Figure 2). Since this effect is potentially important with microglia in the CNS, further studies are essential to elucidate microglial switch during neuroinflammation [2].

### 3.2. Astrocytes

Astrocytes are the most abundant glial cell type in the CNS, which provide mechanical and metabolic support to neurons, and are involved in regulating critical biochemical activities such as neural network, ionic and extracellular space volume homeostasis, synaptic plasticity, and blood flow [62]. In response to a pathological condition, astrocytes change their morphological and functional state and get activated, which could be either beneficial (radial-glial-like astrocytes) or detrimental (reactive astrocytes) as shown in Figure 2 [63].

Upon activation, astrocytes release proinflammatory signaling molecules (e.g., ILs and TNF-*α*), abundantly in the cortex and midbrain [64]. Even though microglia releases inflammatory cytokine at higher level than astrocytes [65], the combined glial response could be essential in the development of neurodegeneration [66]. A dynamic crosstalk between BBB endothelial cells, microglia, astrocytes, and neurons exists and it is expected that a neuroinflammatory response from one cell type will directly impact other [67].

### 3.3. Endothelial Cells

Endothelial cell (EC) is a principal cell type of the BBB. The transport of molecules across the ECs layer is a key to understand how peripheral inflammation can cause prolonged and detrimental brain inflammation. Mediators of inflammation such as cytokines and chemokines were thought to be very large to enter the brain. However, active transport systems were identified at the BBB to allow cytokines movement across the BBB [2, 68]. Humoral factors such as chemokines are associated with the movement of leukocytes across the BBB (Figure 1). For example, CCL19 and CCL21 enable T cell adhesion to the BBB, whereas CXCL12 may play a role in reducing T cell infiltration [69]. Astrocytes produce many such humoral factors, which has effect on the integrity of the BBB. For example, bradykinin induce astrocytes to release of IL-6 to make BBB leaky during inflammation [70]. Furthermore, other cytokines such as IL-1*β* and TNF-*α* were also shown to stimulate permeability of the BBB, enabling the entry of leukocytes in the brain [71]. These cytokines are known to alter BBB integrity by modulating the resistance of tight junctions in brain vasculature ECs [72]. The increased permeability is possibly due to the damage of integral tight-junction proteins (e.g., occludin) through its interaction with the cytoskeleton [73].

### 3.4. Stimulators

DAMPs, also called alarmins and damage signals, are thought to be the principal sterile inflammation triggering agents. These endogenous molecules are recognized by host cells that alert the innate immune system to unscheduled cell death and response to stress. Major putative DAMPs in nonpathogenic neuroinflammation are discussed in a separate section later.

### 3.5. Receptors

Microglia and astrocytes are the major brain cells that express innate immune PRRs like TLRs, RAGE, nod-like receptors (NLRs), scavenger, complement, and mannose receptors. These cells also release cytokines such as TNF, IL-6, IL-1, IFN, and chemokines when stimulated with DAMPs. Major PRRs in astrocytes and microglia are covered in a section later.

### 3.6. Cytokines

Cytokines are proteins of 15–25 kDa molecular weights that have a role of chemical messenger between cells of the immune system. The expression levels of cytokines were elevated in inflammatory conditions such as infection, tissue injury, and immunological alterations and are involved in repairing damaged tissues and restoration of homeostasis [74]. Cytokines are generally classified into pro- and anti-inflammatory cytokines, which facilitate and inhibit inflammatory responses, respectively. IL-1*β*, IL-6, and TNF-*α* are well known proinflammatory cytokines, whereas IL-4 and IL-10 are among the most widely investigated anti-inflammatory cytokines [75].

In the brain, activated microglia and astrocytes are the primary proinflammatory cytokine expressing cells. Under normal physiological conditions, cytokines levels are usually maintained at low levels [76]. However, when infection, trauma, or ischemic attack altered the CNS microenvironment, cytokines expression is activated by glial cells [77]. During pathological conditions, cytokine levels increase 100-fold over normal conditions [75, 78]. Proteins such as lipocalin-2 have secondary function as a cytokine; however, their role in neuroinflammation is still under investigation [79, 80].

### 3.7. Chemokines

Chemokines are small proteins of 8–14 kDa molecular weights that are primarily known for their role of attracting circulating leukocytes to the inflammation or injury sites (Figure 1). Under normal physiological conditions in the brain, chemokines acting on microglia and astrocytes contribute to physiological processes, such as memory, learning, synapse formation, and brain development. Chemokines engage mainly in chemotaxis that are involved in CNS development and homeostatic migration and turnover of cells such as neural precursors in the adult brain [81]. On the other hand during infection or injury, the main change in chemokines is their increased expression level and the most described feature is the chemoattraction of immune cells from the periphery to the brain parenchyma via BBB (Figure 1) [81]. The infiltrated peripheral cells maintain inflammation through cytokine and chemokine secretion, activating resident microglia and astrocytes. Additionally, endothelial cells and neurons were found to constitutively express chemokines and their receptors in the brain. The recruitment and overactivation of such cell types can become deleterious for neuronal survival and function. CX3CL1 or fractalkine/neurotactin, CCL2 or monocyte chemoattractant protein-1, and CXCL12 or stromal cell-derived factor-1 are the three most studied chemokines in the adult CNS [81].

Similar to the neuropeptide and neurotransmitter systems, chemokine system is constitutively and unevenly expressed in the brain with respect to chemokine expressing brain cells [82, 83]. Thus, chemokine system is known to participate in important (patho)physiological processes in the brain, through autocrine or paracrine activity [84]. Moreover, the chemokine system has been shown to interact with neuropeptide and neurotransmitter systems [85, 86].

## 4. Damage-Associated Molecular Patterns

DAMPs are the equivalent of PAMPs but are endogenous molecules. They are vital for tissue repair, whereas they also play important role in the pathogenesis of many inflammatory and autoimmune diseases (Figures 2 and 3). Thus, DAMPs seems to be a double-edged sword [87]. The following are the major characteristics of DAMPs: (1) being released by nonprogrammed cell death such as necrotic, aponecrotic, necroptosis, and pyroptosis; (2) being released by immune cells without dying, which are generally secreted by endoplasmic reticulum-golgi secretion pathway; (3) activating receptor-expressing cells of the immune system and thus directly or indirectly promoting innate or adaptive immune responses; (4) regulating the inflammatory response to clear injury and initiate repair; however, excessive activation of inflammation may cause further damage. A putative list of DAMPs recognized in the CNS inflammation is provided in Table 1, and some are discussed below.

### 4.1. High-Mobility Group Box  1 Protein

HMGB1 is a DNA-binding protein that is widely expressed in various tissues including brain. The release of HMGB1 in the extracellular milieu from damaged neurons and oligodendrocyte-like cells serves as damage signal to evoke inflammatory reactions, such as the activation of endothelial cells, glial cells, and various blood immune cells, exacerbating brain damage (Figures 1–3) [88, 89].

Elevation of HMGB1 in brain was measured in nondegenerative neuroinflammatory condition such as TBI [90], ethanol exposure [91], and stress-induced neuroinflammatory priming [92]. In addition, HMGB1 was discovered to be released from neurons and glia in a mouse model of acute and chronic seizures [20]. Further, its level is significantly high in the cytosolic and particulate fractions of AD brains [93]. Also, HMGB1 seems to colocalize with A*β* in senile plaque that are associated with activated microglia, inhibiting microglial clearance and enhancing A*β* neurotoxicity [93, 94]. A neuropathological hallmark of PD is the abnormal accumulation of *α*-synuclein filaments in Lewy bodies. Several studies indicated the preferential binding of HMGB1 to aggregate *α*-synuclein in Lewy bodies [95, 96]. Further, in animal models of PD, an interaction between a microglial PRRs, Mac1, and HMGB1 was identified. The HMGB1-Mac1-NADPH oxidase signaling axis is known to induce chronic inflammation and progressive dopaminergic neurodegeneration, indicating the possible role of persistent inflammation and chronic neurodegeneration [94, 97, 98]. Interestingly, HMGB1 seems to be neuroprotective against the polyglutamine repeats toxicity in the HD models by exhibiting chaperone-like activity [99].

Studies indicate that HMGB1 acts as a ligand for RAGE, TLR2, and TLR4, which ultimately activates several MAPKs and NF-*κ*B to regulate the expression of classic proinflammatory cytokines such as IFN*γ*, IL-1*β*, IL-1*α*, TNF-*α*, and IL-6 [100, 101]. Altogether, the role of HMGB1 in brain is less than straightforward. However, in all diseases and animal models it has capacity to assume a proinflammatory role. Understanding the function of HMGB1 and its receptors in different contexts is important in positioning it as a potential therapeutic target for neuroinflammatory conditions.

### 4.2. Heat Shock Proteins

HSP is a family of molecular chaperones that facilitate the stabilization of damaged polypeptides. There are six major subfamilies: HSP100, HSP90, HSP70, HSP40, and small HSPs (e.g., HSP27 and *α*B crystalline) [94]. The role of HSPs as DAMPs in brain injury has not yet been fully elucidated. However, there are multiple reports implicating HSPs in various tissue injury models [102, 103]. These studies demonstrated the extracellular release of HSPs from injured cells, activating inflammation in surrounding cells [104].

HSP70 present in the extracellular milieu was shown to bind TLR2 and TLR4 in the inflammatory cells and induce the expression and release of cytokines [105]. In a more recent study, intrathecally injected HSP60 injured neuronal cells and oligodendrocytes in the CNS, whereas mice lacking TLR4 and MyD88 (TLR4 adaptor molecule) are found to be protective against deleterious effects of HSP60 [106]. In an animal model of stroke, namely, middle cerebral artery and reperfusion (MCAO), inhibition of HSP90 by 17-dimethylaminoethylamino-17-demethoxygeldanamycin (17-DMAG) protected BBB integrity [107].

HSP70 induced in TBI had protective effects against brain injury, suggesting its pharmacological role [108]. Further, heat stress-induced HSP70 rendered neuroprotection by interrupting the phosphorylation of I*κ*B, JNK, and p38 in astrocytes, effectively downregulating the expression of proinflammatory genes [109]. In neurodegenerative diseases, including AD, HSP70 is thought to induce protein conformational change in favor of nontoxic form [110]. In a separate study, both animals and human patients of temporal lobe epilepsy (TLE) had high HSP60 in their plasma and hippocampus; thus it has also been proposed as a biomarker of hippocampal stress having potential use for the diagnosis and TLE management [111]. Altogether, these reports indicate diverse function of HSPs depending on their type and disease condition. Since HSPs bind to misfolded proteins to assist the correct folding, it is not unlikely that the role of HSPs as DAMPs was misunderstood for the proteins with altered structure. Further extensive studies are essential to test this hypothesis.

### 4.3. S100B

Calcium-modulate protein B (S100B) is a member of S100 super family that is primarily secreted from astrocytes [112]. It is neurotropic in the nanomolar concentration but has lethal effects on neuronal integrity in micromolar doses. Also, at higher doses, S100B promotes neuroinflammation via the activation of RAGE in astrocytes and microglia [113]. In AD, the level of S100B is highest in the most severely affected regions of the brain, being associated with plaques [114]. Additionally, AD patients with higher S100B levels exhibit lower cognitive scores [115]. In mouse model of AD, S100B expression promotes A*β* biogenesis and tau hyperphosphorylation, enhancing the neuroinflammation [116]. Also, the pharmacological inhibition of S100B expression by arundic acid ameliorates plaque load and gliosis in the cortex and hippocampus [117].

In the brain of PD patients, S100B protein level is highly elevated in the degenerating substantia nigra region [118]. S100B gene ablation in mice protected them against 1-methyl-4-phenyl-1,2,3,6-tetrahydropyridine- (MPTP-) induced neurotoxicity via the reduction in microgliosis and expression of RAGE and TNF-*α* receptor [118]. In HD, RAGE colocalizes with S100B, especially in astrocytes, which is thought to impact the HD progression via the activation of NF-*κ*B [119]. However, some studies predicted neuroprotective role of S100B [120]; thus, the verdict regarding beneficial or detrimental role of S100B in PD remains open. S100B level is also higher in the brain and blood of patients suffering from epilepsy and TBI [121, 122]. Due to the constant elevation of S100B in the serum of neuroinflammatory conditions with compromised BBB, it is considered as a marker of BBB integrity [123, 124].

The extracellular role of other members of S100 superfamily such as S100A8 and S100A9 has been studied in peripheral acute/chronic inflammatory disorders. However, their expression and functions in brain remain enigmatic.

### 4.4. Deoxyribonucleic Acid

DNA is tightly packed in the nucleus. However, freely circulating DNA (both nuclear and mitochondrial) was detected in the plasma of critically ill and old myocardial infarction and trauma patients [125, 126]. In a study of 800 Caucasian subjects, Pinti et al. measured increased plasma levels of mt-DNA after the 5th decade of life [126]. During aging, proinflammatory cytokines such as TNF-*α*, IL-6, IL-1*β*, and RANTES are mildly elevated in the plasma, which is recognized as “inflammaging” [126–128]. More recent study showed the induction of proinflammatory cytokines secretion in primary astrocytes by mt-polynucleotides [129]. In vitro, mt-DNA induced the expression of cytokines via TLR9 in monocytes [126]. This DNA sensing receptor is highly expressed in the astrocytes and microglia, suggesting its potential role in neuroinflammation [128].

Cell-free DNA is thought to arise from necrotic or apoptotic cells. Similar to mt-DNA, the quantity of nuclear cell-free DNA seems to be associated with aging [125]. TLR9 is the ubiquitous receptor for the endocytosed DNA. However, it is also thought to activate non-TLRs, expanding the list of DNA sensing receptors and downstream pathways [130]. Further studies are essential for a clear understanding on the roles of DNA (and RNA) in neuroinflammation and neurodegeneration.

### 4.5. Adenosine Triphosphate

ATP is a purine base that mediates biochemical processes such as glucose metabolism, biosynthesis, and muscle contraction within the cell. Even though ATP has role inside the cell, it is released extracellularly from injured or dead cells and triggers the activation of NLRP3, P2X7R, and caspase-1 to release inflammatory mediators such as IL-6, TNF-*α*, and COX-2 [131, 132]. In a more recent study, mitochondrial lysates induced inflammation and showed AD-like changes in microglial and neuronal cells, indicating the potential role of extracellular ATP in neuroinflammatory conditions [133]. Additionally, extracellular ATP significantly increased the intracellular *α*-synuclein levels in neurons, causing lysosomal dysfunction [134]. The accumulation of *α*-synuclein in neurons to form Lewy bodies is the pathological hallmark of PD.

Extracellular ATP is thought to be toxic for primary neuronal as well as organotypic CNS cultures [94]. In BV2 microglial cells, neuronal mitochondrial lysates induced the expression of TNF-*α*, NF-*κ*B, and IL-8 mRNA and phosphorylation of p38 MAPK [133]. Additionally, emerging studies showed that pharmacological targeting of ATP-gated purinergic P2 receptors (P2X1-7 and P2Y11) can potentially modulate the generation of seizures, seizure-induced brain damage, and inflammatory processes [135–137]. Collectively, extracellular ATP has DAMP-like function, triggering neuroinflammation and elevating neurodegeneration.

### 4.6. Uric Acid

Uric acid is the ultimate catabolite of purine metabolism, which is disposed via kidneys and excreted in urine. Also, it is a main antioxidant in blood [138]. Uric acid in the PD model considerably attenuated the disease [139]. A study in AD patients also showed protective role of uric acid [140]. Exogenous administration of uric acid is also neuroprotective in experimental models of CNS diseases such as brain ischemia, meningitis, and ischemic stroke [138]. A more recent study in stroke patients showed that decrease in blood uric acid levels during the first week after onset of stroke correlated to more severe stroke, unfavorable stroke evolution, and poor long-term stroke outcome [141].

The deficiency in uricase enzyme increases serum levels of uric acid that forms monosodium urate (MSU) crystals. Also, when extracellular uric acid comes in contact with high levels of free sodium, it is believed to nucleate and form MSU crystals [142]. These MSU crystals are sensed by IL-1R, TLR2, and TLR4, which then activate NLRP3 inflammasome leading to IL-1*β* production [143, 144]. Taken together, microenvironment of uric acid may determine whether it has therapeutic or toxic effects. The pathological mechanisms of MSU crystal formation and NLRP3 inflammasome/caspase-1 activation in the brain remain to be addressed.

## 5. Pattern-Recognition Receptors

A group of receptors that are engaged to recognize certain molecular structures or patterns are referred to as PRRs. Since these receptors sense multiple molecules (PAMPs and DAMPs) to induce downstream signaling, they do not follow the classical 1 : 1 ligand-receptor relation [79, 145]. The role of activation of these PRRs is to protect host against danger, but their aberrant activation could contribute to accelerate inflammatory processes (Figure 4). Some of the major PPRs are discussed below.

### 5.1. Toll-Like Receptors

TLRs comprise a large family of transmembrane receptors that recognize a diverse range of exogenous or endogenous molecular signals, activating the innate immune system [146]. TLR2 and TLR4 are membrane bound surface receptors that sense extracellular DAMPs, whereas TLR9 is located intracellular that sense intracellular DAMP ligand such as DNA [94]. Interestingly, peripheral tissue damage (e.g., following cytotoxic treatment) also possesses TLR-mediated glial activation capacity [147, 148]. Moreover, TLRs involved in signaling DAMPs may interact at several levels, but almost all converge into the activation of NF-*κ*B [149].

TLR4 is the most extensively characterized TLR subtype with established host immune response. Until recently, TLR4 expression in the brain was limited to microglia, astrocytes, and oligodendrocytes. New studies have now shown that TLR4 is also expressed on CNS structures such as choroid plexus, circumventricular organs, and leptomeninges [150]. Recent evidences have linked TLR4-signaling in multiple neurodegenerative conditions such as AD, PD, stroke, and TBI [151, 152]. When activated, TLR4 recruits adaptor molecules and kinases, initiating the downstream signaling cascade that ends with the secretion of proinflammatory cytokines and chemokines [151]. TNF, IL-1*β*, IL-1 receptor, IL-6, IL-8, IL-10, IL-12p40, IL-23, MIP-1*α*/1*β*, IFN-*β*, and chemokines are downstream product of TLR4 pathway [153]. These factors induce inflammatory reactions within the CNS and facilitate the inflammatory response by increasing vascular permeability, directing dendritic cells, and initiating macrophage entry into the CNS (Figures 1–4) [154]. Other TLRs, primarily TLR2 and TLR9, were also stimulated in multiple neuroinflammatory conditions (review, TLRs in AD [155]). Together, studies suggest TLRs as a potential robust drug target to ameliorate severe neuroinflammation.

### 5.2. Receptor for Advanced Glycation End Products

RAGE belongs to the immunoglobulin superfamily, which is expressed in astrocytes, microglia, neurons, and endothelial cells in brain. It was recognized as a receptor for advanced glycation end products (AEGs). However, RAGE interacts with a variety of other endogenous ligands such as A*β*, HMGB1, and S100, and exogenous molecules of bacteria and prions. Stimulation of RAGE activates MAPKs (e.g., extracellular signal-regulated kinases 1/2, Erk1/2; p38 MAPK) and NF-*κ*B [94, 156].

### 5.3. NOD-Like Receptors

NLRs are expressed in several immune and nonimmune cells that sense variety of PAMPs and DAMPs intracellularly. Pyrin domain containing receptors (NLRPs), leucine reach repeat, and nucleotide-binding oligomerization domain belong to this receptor family [94, 157]. Inflammasome-forming NLRs are the extensively studied and well characterized classes of NLRs. The signal specificity and functional roles of inflammasome-forming NLRs are not yet known clearly. Upon sensing PAMPs or DAMPs, an NLR is thought to forms a multimeric protein complex called inflammasome. It is a large macromolecular complex that contains multiple copies of pattern recognizing receptors, caspase-1, and an adaptor protein called apoptosis-associated speck-like protein containing a caspase recruitment domain (ASC) [158, 159]. As shown in Figure 4, caspase-1 then mediates the cleavage of pro-IL-1*β* and pro-IL-18 into their mature forms of IL-1*β* and IL-18, which has critical roles in mediating immune responses during inflammation and innate immunity [158]. Stimulation of NLRs also promotes downstream activation of NF-*κ*B or MAPK signaling pathways, leading to the increase in production of cytokines and chemokines [94].

## 6. Therapeutic Strategies to Alleviate Sterile Neuroinflammation

**Figure 5:**
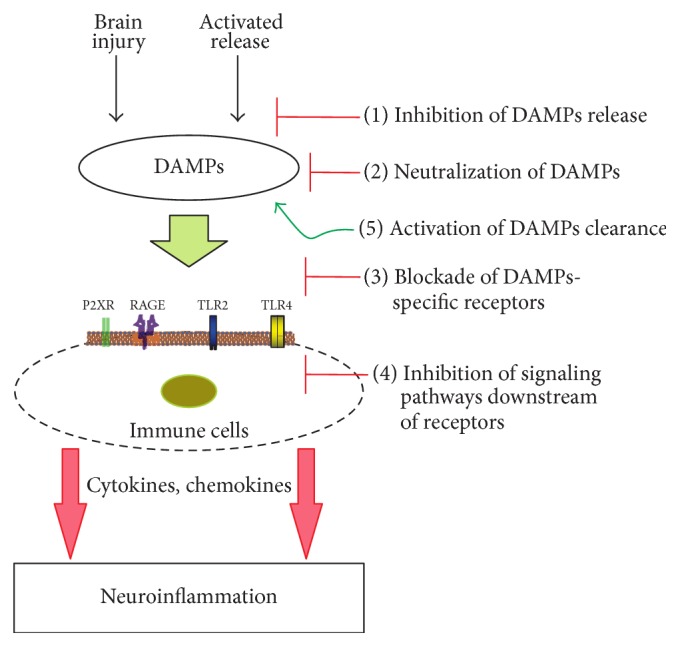
Drug treatment strategies for DAMPs-induced neuroinflammation. Preclinical studies have identified a number of multipotential drug targets that attenuates neuroinflammation triggered by DAMPs released after brain injury or excessive neurodegeneration.

In the physiological conditions, DAMPs are essential to initiate tissue repair. However, release of enormously large amount of DAMPs and uncontrolled activation of PRRs contribute to the pathogenesis of many neuroinflammatory conditions (Figure 3). The possibilities and attempts of targeting DAMPs, its receptors, and other downstream signaling molecules to attenuate excessive neuroinflammation are discussed below (Figure 5). HMGB1 and its receptors are mostly targeted in several inflammatory conditions, which are also reflected in this review. A list of drugs that are able to attenuate nonpathogenic CNS inflammation are provided in Table 2.

### 6.1. Inhibition of DAMPs Release

Endogenous neuropeptides, specifically vasoactive intestinal peptide (VIP) and urocortin, acted as inhibitors of HMGB1 cytokine activity that increased the survival of animals with established endotoxemia. Additionally, another endogenous neuropeptide, pituitary adenylate cyclase-activating polypeptide (PACAP), significantly reduced circulating HMGB1 levels and rescued animals in lethal endotoxemia administration [160]. In mice, these neuropeptides downregulated the translocation of HMGB1 from nucleus into the cytoplasm; this protective effect was completely reversed by the administration of recombinant HMGB1 [160, 161].

Acetylcholine, neurotransmitter, is shown to inhibit HMGB1 release from human macrophages by signaling through a nicotinic acetylcholine receptor (nAChR). The stimulation of nAChRs inhibited HMGB1 secretion induced by TNF-*α* via NF-*κ*B activation [162]. The blockade of glutamate/NMDA stimulation by MK-801 prevented the release of HMGB1 inhibiting neuroinflammation through TLR4 [163]. Eicosapentaenoic acid (EPA), a peroxisome proliferator-activated receptor gamma (PPAR*γ*) agonist, restored the optimum PPAR*γ* expression, attenuating the ischemic brain damage by downregulating the release of HMGB1 signal related molecules [164].

A major constituent of licorice root, glycyrrhizin (GL), is suggested to inhibit the release of HMGB1 in TBI rat and mouse models. GL suppressed the BBB permeability and impairment of motor functions along with the inhibition of HMGB1 translocation in the neurons at injury sites [165]. In MCAO model of stroke, i.v. administration of GL significantly reduced infarct volumes, showing neuroprotection via anti-inflammatory property by inhibiting HMGB1 secretion [161, 166]. Carbenoxolone (CBX) is a synthetic GL derivative that abrogated lipopolysaccharide- (LPS-) induced HMGB1 release in macrophage cultures by impairing PKR activation [167].

A Chinese herb “Danshen” contains the abundant red pigments called tanshinones that has similar structure to steroidal anti-inflammatory drugs. These steroid derivatives significantly protected mice against lethal endotoxemia by selectively blocking the endotoxin-induced cytoplasmic translocation and release of HMGB1 [168]. Two statin molecules, atorvastatin and simvastatin, protected rat brains from ischemic injury by significantly attenuating the overexpression of HMGB1, RAGE, TLR4, and NF-*κ*B induced in ischemia [169].

Synthetic molecules such as nafamostat mesilate (NM), gabexate mesilate (GM), and sivelestat sodium hydrate drastically reduced LPS-induced injury at least partly by inhibiting HMGB1 [170–172]. Ethyl pyruvate (EP) inhibited nuclear-to-cytoplasmic translocation of HMGB1 and markedly attenuated the expressions of TLR4, DNA-binding activity of NF-*κ*B, and expression of inflammatory mediators (IL-1*β*, TNF-*α*, and IL-6) in TBI rats [173, 174].

### 6.2. Neutralization of DAMPs

Emerging evidences support that HMGB1 neutralizing antibodies and HMGB1 A box antagonist have therapeutic potential to ameliorate excessive inflammation [175]. HMGB1 antagonist competitively inhibits HMGB1 surface binding and attenuates proinflammatory cytokine release in stroke [176, 177]. It efficiently interacts with RAGE, competing with the binding of full-length protein, but does not activate the receptor, lacking the proinflammatory activity located on the box  B [161, 178].

In a rat model, anti-HMGB1 mAb significantly prevented dopaminergic neurons in SNPC and dopaminergic terminals in the striatum to attenuate behavioral symptoms of PD. In addition, intravenously administered anti-HMGB1 mAb suppressed BBB disruption and neuroinflammation [179]. In glutamate/NMDA excitotoxicity model of neurodegeneration, HMGB1 neutralizing antibodies block neuroimmune induced neuronal death [163]. Further, neutralization of DAMPs was predicted to enhance the clearance of A*β* in AD patients [93]. Orally active vaccine developed to neutralize pathogenic effect of A*β* has also improved cognitive function of AD mice [180]. Treatment with neutralizing antibody was associated with less phosphorylation of I*κ*B, which successfully reduced damage caused by ischemia reperfusion injury in mice [181].

DAMPs such as HSPs perhaps are not the good therapeutic targets, because of their critical role in the cell survival. Thus, it is essential to understand altered structures of HSPs that are alarmins so that only structurally altered HSPs can be targeted by neutralizing antibodies or antagonists. The idea of using anti-DAMP antibodies is mainly to reduce pathological conditions and augment the efficacy of potential therapeutic approaches based on its blockade. Antibody-based strategy suffers from a multiple drawback of possible conformational switches in the tertiary structure of the antibody-recognition sites. Furthermore, humanization of antibodies is another challenge for the development of anti-DAMP antibodies.

### 6.3. Blockade of DAMPs-Specific Receptors

A study was carried out to investigate immune response in AD model by neutralization of TLR2 and TLR4 using anti-TLR2 and anti-TLR4 antibodies. TLR2 and TLR4-specific antibodies blocked ~50% and ~30% of cell response to trigger innate immunity to fibrillar A*β*(1-42) [182]. To neutralize a receptor of A*β*, RAGE, an orally active aqueous vaccine against a RAGE/A*β* complex was developed. In vitro prepared RAGE/A*β* complex induced a greater immunogenic response in both human and mice than individual RAGE or A*β*. Further, an orally administered vaccine of RAGE/A*β* complex or A*β* alone improved the cognitive function of AD transgenic mice. Also, RAGE/A*β* complex is more effective than A*β* [180].

S100P-derived small peptides blocked RAGE-mediated signaling at micromolar concentration, quenching NF-*κ*B activity. These peptides inhibited glioma tumor growth by reducing the ability of these ligands to stimulate RAGE [161, 183]. N-Benzyl-4-chloro-N-cyclohexylbenzamide (FPS-ZM1) is a biologically nontoxic and BBB permeable RAGE specific antagonist that attenuated neuroinflammation in AD [184] and subarachnoid hemorrhage (SAH) animal models [185]. Since many DAMPs signal inflammation induction via RAGE, FPS-ZM1 potentially dampens inflammation induction in other neuroinflammatory conditions. More recently, a newly developed, potent, and selective inhibitor of NLRP3, MCC950, significantly inhibited IL-1*β* production in an animal model of multiple sclerosis, which could be a potential therapeutic agent for other NLRP3-associated neuroinflammatory conditions [186]. Also, inhibitors of proinflammatory cytokines such as IL-1 receptor antagonists potentially attenuate the propagation of neuroinflammation [187].

It is important to note that the complete blockade of PRRs would be detrimental for cell survival. A study in mice showed that the absence of TLR2 impaired hippocampal neurogenesis. Further, TLR2 and TLR4 directly modulated self-renewal and the cell-fate decision of neural stem progenitor cells [188].

### 6.4. Inhibition of Signaling Pathways Downstream of Receptors

Resveratrol is a natural polyphenol associated with anti-inflammatory effects by preferentially inhibiting NF-*κ*B activation following cytokine release upon A*β* stimulation. Resveratrol is currently in clinical trials for AD, which significantly decreased microglial activation and lowered cerebral amyloid deposition in the animal model of AD [189]. Exogenous PACAP inhibited the upregulation of TLR4 and its downstream signaling molecules MyD88, P-I*κ*B, and NF-*κ*B in TBI animal model, which ultimately suppressed the expression if inflammatory agents such as IL-1*β* and TNF-*α* in the brain. In addition, PACAP significantly improved motor and cognitive dysfunction, decreased brain edema, and reduced neuronal cell death following TBI [190]. Methotrexate (MTX) is used in chemotherapy of tumors and autoimmune diseases, which was identified to directly interact with HMGB1. The binding of MTX inhibited HMGB1/RAGE interaction at molecular and cellular levels, reducing the anti-inflammatory function of HMGB1 [191].

A cholesterol-lowering agent, simvastatin, has demonstrated neuroprotective effect by markedly attenuating the expression of TLR4, NF-*κ*B, and downstream inflammatory modulator (e.g., IL-1*β*, IL-6, TNF-*α*, and ICAM-1) after TBI in rats [192]. Tanshinone II A (Tan IIA) markedly reduced the expression levels of HMGB1, TLR4, RAGE, and NF-*κ*B after ischemia in rats [193]. Luteolin, present in various fruits and vegetables, has the ability to downregulate TLR4 and NF-*κ*B expression and protect rat against the focal ischemia [194]. MLN519 is a well characterized proteasome inhibitor, which also has a role to modulate NF-*κ*B activity, attenuating expression of cytokines and cellular adhesion molecules and reducing neutrophil and macrophage infiltration into the ischemic rat brain [195].

### 6.5. Activation of DAMPs Clearance

During the normal physiological conditions, potential DAMPs can be eliminated by several mechanisms. For example, A*β* can be degraded by enzymes neprilysin and insulin-degrading enzyme in the brain parenchyma [196], absorbed into the blood by low density lipoprotein receptor protein-1 pathway [197], and cleared by perivascular lymphatic drainage pathways [198].

Drainage of the brain extracellular fluids, particularly interstitial fluid (ISF) and CSF, is important for volume regulation. However, recent evidences suggest its role for the removal of waste products (e.g., p-tau and A*β*), which is thought to be imbalanced in neurodegenerative diseases [199, 200]. The abnormal phosphorylation of protein such as tau is a contributing factor to the pathogenic processes to a toxic gain of function (e.g., increased tau-tau, tau-A*β*, and interaction), making it difficult to eliminate [201].

Methylthioninium chloride (MTC) is a first identified tau aggregation inhibitor that facilitates the clearance of abnormally phosphorylated tau [202]. Identification of tau specific (hyper)phosphorylation inhibitor would be extremely beneficial to enhance the elimination of abnormally folded protein. In addition, adding a tag to the tangled (e.g., p-tau), structurally altered (e.g., HMGB1, HSPs, and S100B), and aggregated proteins (e.g., A*β*) would improve the lymphatic clearance of brain by increasing their solubility in ISF and CSF.

## 7. Concluding Remarks

Current strategies in clinical development to attenuate detrimental effects of neuroinflammation include (1) global blockade of DAMP receptors such as TLRs and RAGE using natural antagonists, small molecule inhibitors, soluble receptor-specific extracellular domains (e.g., TLR extracellular domains), and neutralizing antibodies; (2) inhibition of signaling pathways downstream of DAMP receptors stimulation such as MyD88/TRAF/IRAK complex formation, MAPK or I*κ*B phosphorylation, and NF-*κ*B translocation using small molecules.

The global blockage of PRRs might create a problem by suppressing immune response essential during pathogen invaded infection. Thus, a comparative analysis of downstream signaling domains such as transcription factors, adaptors, and kinases activated by PAMPs versus DAMPs is essential to highlight key differences, because, if selectively targeted, it could lead to specific therapies engineered to silence danger signals without compromising host immune defense.

The idea of targeting inflammatory activities of DAMPs to confer protection against tissue injury is validated in multiple preclinical studies. The discovery of drug targets to ameliorate brain injury by neutralizing DAMPs, activating DAMPs clearing processes, and inhibiting DAMPs release provides a new paradigm for the strategic development of experimental therapeutics. The emerging research field deserves to be largely explored also in the search for effective drugs to attenuate uncontrolled neuroinflammation triggered by DAMPs.

## Figures and Tables

**Table 1:** A putative list of DAMPs.

DAMPs	Neurological conditions	DAMPs releasing cells	Target cells	Target receptors	Downstream expression	References
HMGB1	Brain ischemia, TBI, stroke, ethanol exposure, AD, PD, HD	Injured neurons, oligodendrocyte-like cells, glial cells	Microglia, astrocytes, blood vessel-associated cells	RAGE, TLR2, TLR4	IL-1*β*, IFN*γ*, IL-1*α*, TNF*α*, IL-6	[20, 40, 88, 89, 92–94]

HSPs	TBI, stroke	Injured cells	Microglia	TLR2, TLR4, RAGE	TNF*α*, IL-1*β*, ICAM-1, VCAM-1, E-selection, iNOS	[105–107, 203]

S100B	AD, PD, HD	Astrocytes	Astrocytes, microglia	RAGE	Bcl-2 (antiapoptotic)	[112, 113]

DNA	Aging, TBI	Damaged or dead neurons	Astrocytes, microglia	TLR9	TNF*α*, IL-1*β*, RANTES	[125–129]

ATP	Stroke, PD, seizure	Damaged mitochondria	Microglia	NLRP3, P2X7RRAGE	TNF*α*, IL-6, COX-2, IL-8	[30, 131–133, 135, 136]

*HMGB1*, high mobility group box 1 protein; *HSPs*, heat shock proteins; *S100B*, calcium-modulated proteins B; *DNA*, deoxyribonucleic acid; *ATP*, adenosine triphosphate; *TBI*, traumatic brain injury; *AD*, Alzheimer's disease; *PD*, Parkinson's disease; *HD*, Huntington's disease; *RAGE*, receptor for advanced glycation end products; *TLR*, toll-like receptor; *IL*, interleukin; *TNF*, tumor necrosis factors; *IFN*, interferon; *ICAM*, intercellular adhesion molecule; *VCAM*, vascular cell adhesion molecule; *iNOS*, inducible nitric oxide synthase; *Bcl-2*, b-cell lymphoma 2; *RANTES*, regulated on activation, normal T-cell expressed and secreted; *COX-2*, cyclooxygenase-2.

**Table 2:** A list of drugs that ameliorate DAMPs-induced neuroinflammation.

Mode	Drug	Remarks	References
Inhibition of DAMPs release	VIP, urocortin, PACAP, acetyl choline	Endogenous, inhibiting nucleus-to-cytoplasm transport of HMGB1	[160–162]
	EP	Inhibiting nucleus-to-cytoplasm transport of HMGB1	[173, 174]
	MK-801	Blocking glutamate/NMDA receptor, reducing HMGB1 release	[163]
	EPA	PPAR*γ* agonist, attenuating HMGB1 release	[164]
	GL	Constituting licorice root, inhibiting HMGB1 release	[161, 165, 166]
	CBX	Synthetic GL, reducing HMGB1 secretion	[167]
	Tanshinones	Constituting herb “Danshen”, inhibiting HMGB1 release	[168]
	Atorvastatin, simvastatin	Attenuating the extracellular release of HMGB1	[169]
	NM, GM, sivelestat	Synthetic, inhibiting HMGB1 secretion	[170–172]

Neutralization of DAMPs	HMGB1 A box	HMGB1 lacking proinflammatory activity, competitively inhibits HMGB1	[161, 175–177]
	Anti-HMGB1 antibody	Reduction in proinflammatory role	[179]
	A*β* vaccine	Generation of anti-A*β* antibodies	[180]

Blockade of DAMPs-specific receptors	VIPER	Peptide, blocking TLR4	[204]
	Anti-TLR2 antibody	Reduction in proinflammatory role	[182]
	Anti-TLR4 antibody	Reduction in proinflammatory role	[182]
	RAGE vaccine	RAGE/A*β* complex has greater immunogenic response than RAGE or A*β* alone	[180]
	S100P-derived peptide	Competitive inhibitor of RAGE	[161, 183]
	FPS-ZM1	RAGE specific antagonist	[184]
	MCC950	Potent, selective inhibitor of NLRP3	[186]
	IL-1RA	Blocking IL-1R	[187]

Inhibition of signaling pathways downstream of receptors	Resveratrol	Natural polyphenol	[189]
	Exogenous PACAP	Inhibiting the upregulation of TLR4, MyD88, P-I*κ*B, and NF-*κ*B	[190]
	MTX	HMGB1 binding property, reducing HMGB1/RAGE interaction	[191]
	Simvastatin	Attenuating TLR4 and NF-*κ*B expression	[192]
	Luteolin	Fruit and vegetable constituent, downregulating TLR4 and NF-*κ*B	[194]
	Tan IIA	Reducing the expression of HMGB1, TLR4, RAGE, and NF-*κ*B	[193]
	MLN519	Protease inhibitor, modulating NF-*κ*B activity	[195]

Activation of DAMPs clearance	MTC	Inhibiting tau aggregation	[202]

*VIP*, vasoactive intestinal peptide; *PACAP*, pituitary adenylate cyclase-activating polypeptide; *EP*, ethyl pyruvate; *EPA*, eicosapentaenoic acid; *PPARγ*, peroxisome proliferator-activated receptor gamma; *GL*, glycyrrhizin; *CBX*, carbenoxolone; *NM*, nafamostat mesilate; *GM*, gabexate mesilate; *VIPER*, viral inhibitory peptide; *FPS-ZM1*, n-benzyl-4-chloro-N-cyclohexylbenzamide; *IL-1RA*, IL-1 receptor antagonist; *MTX*, methotrexate; *Tan IIA*, tanshinone II A; *MTC*, methylthioninium chloride.

## Abbreviations

AD: Alzheimer's disease
AGEs: Advanced glycation end products
ALS: Amyotrophic lateral sclerosis
ASC: Apoptosis-associated speck-like protein
ATP: Adenosine triphosphate
A*β*: Amyloid beta
BBB: Blood-brain barrier
BBBD: BBB damage
CBX: Carbenoxolone
CNS: Central nervous system
COX: Cyclooxygenase
CSF: Cerebrospinal fluid
DAMPs: Damage-associated molecular patterns
DNA: Deoxyribonucleic acid
EP: Ethyl pyruvate
EPA: Eicosapentaenoic acid
FPS-ZM1: N-Benzyl-4-chloro-N-cyclohexylbenzamide
GL: Glycyrrhizin
GM: Gabexate mesilate
HD: Huntington's disease
HIV: Human immunodeficiency virus
HMGB1: High-mobility group box1 protein
HSPs: Heat shock proteins
ICAM: Intracellular adhesion molecule
IFN: Interferon
IL: Interleukin
iNOS: Inducible nitric oxide synthase
IRAK: Interleukin-1 receptor-associated kinase
ISF: Interstitial fluid
I*κ*B: Inhibitor of kappa B
JNK: c-Jun N-terminal kinase
LPS: Lipopolysaccharides
mAb: Monoclonal antibody
MAL: MyD88-adaptor like
MAPK: Mitogen-activated protein kinase
MCAO: Middle cerebral artery and reperfusion
MCP: Monocyte chemoattractant protein
MIP: Macrophage inflammatory protein
MPTP: 1-Methyl-4-phenyl-1,2,3,6-tetrahydropyridine
MSU: Monosodium urate
MTC: Methylthioninium chloride
MTX: Methotrexate
MyD88: Myeloid differentiation primary response gene (88)
nAChR: Nicotinic acetylcholine receptor
NADPH: Nicotinamide adenine dinucleotide phosphate
NF-*κ*B: Nuclear factor-kappa B
NLRPs: Pyrin domain containing receptors
NLRs: Nod-like receptors
NMDA: N-Methyl-D-aspartate
P2XR: ATP-gated purinergic P2 receptors
PACAP: Pituitary adenylate cyclase-activating polypeptide
PAMPs: Pathogen-associated molecular patterns
PD: Parkinson's disease
PKR: Protein kinase R
PPAR*γ*: Peroxisome proliferator-activated receptor gamma
PRRs: Pattern-recognition receptors
PTSD: Posttraumatic stress disorder
RAGE: Receptor for advanced glycation end products
SAH: Subarachnoid hemorrhage
SNPC: Substantia nigra pars compacta
Tan IIA: Tanshinone II A
TBI: Traumatic brain injury
TGF: Transforming growth factor
TLE: Temporal lobe epilepsy
TLRs: Toll-like receptors
TNF: Tumor necrosis factor
TRAM: TRIF-related adaptor molecule
TRIF: TIR-domain-containing adapter-inducing interferon-*β*
VCAM: Vascular cell adhesion molecule
VIP: Vasoactive intestinal peptide
VIPER: Viral inhibitory peptide.
